# Impact of Caregiver Type for 3-Year-Old Children on Subsequent Between-Meal Eating Habits and Being Overweight From Childhood to Adulthood: A 20-Year Follow-up of the Ibaraki Children’s Cohort (IBACHIL) Study

**DOI:** 10.2188/jea.JE20140078

**Published:** 2015-09-05

**Authors:** Mizuki Sata, Kazumasa Yamagishi, Toshimi Sairenchi, Ai Ikeda, Fujiko Irie, Hiroshi Watanabe, Hiroyasu Iso, Hitoshi Ota

**Affiliations:** 1 Public Health, Department of Social Medicine, Osaka University Graduate School of Medicine, Suita, Osaka, Japan; 1 大阪大学大学院医学系研究科社会医学講座公衆衛生学; 2 Ibaraki Health Plaza, Ibaraki Health Service Association, Mito, Ibaraki, Japan; 2 公益財団法人 茨城県立総合健診協会 茨城県立健康プラザ; 3 Department of Public Health Medicine, Faculty of Medicine, University of Tsukuba, Tsukuba, Ibaraki, Japan; 3 筑波大学医学医療系社会健康医学; 4 Department of Public Health, Dokkyo Medical University School of Medicine, Mibu, Tochigi, Japan; 4 獨協医科大学医学部公衆衛生学講座; 5 Department of Public Health, Juntendo University School of Medicine, Tokyo, Japan; 5 順天堂大学大学院医学研究科公衆衛生学講座; 6 Department of Health and Welfare, Ibaraki Prefectural Office, Mito, Ibaraki, Japan; 6 茨城県保健福祉部保健予防課; 7 Ibaraki Health Service Association, Mito, Ibaraki, Japan; 7 公益財団法人 茨城県総合健診協会

**Keywords:** children, eating habits, overweight, cohort study, epidemiology

## Abstract

**Background:**

Because of the increasing number of mothers who continue to work after childbirth, participation in childcare has diversified. However, the impact of the main caregiver on children’s habits has not been determined. We sought to examine the effect of caregiver differences on childhood habituation of between-meal eating and body mass index (BMI).

**Methods:**

The Ibaraki Children’s Cohort Study involved 4592 Japanese children whose parents answered health questionnaires at age 3. Follow-up questionnaires were distributed to parents when children were 6 and 12 years old and to study subjects directly when they were 22 years old. We compared prevalence of between-meal eating and overweight as well as mean BMI at ages 6, 12, and 22 years, by their main daytime caregiver at age 3.

**Results:**

Compared to children cared for by mothers, those cared for by grandparents had a higher prevalence of between-meal eating before dinner for boys and girls at ages 6 and 12 years. At age 22 years, boys cared for by grandparents had a higher prevalence of overweight than those cared for by mothers (18.5% versus 11.2%, *P* = 0.037), but no such difference was noted in girls. However, both boys and girls cared for by grandparents had higher mean BMI over time than those cared for by mothers (coefficient = 0.47 kg/m^2^ for boys and coefficient = 0.35 kg/m^2^ for girls).

**Conclusions:**

Being cared for by grandparents at age 3 was associated with subsequent between-meal eating habits, being overweight, and increased mean BMI from childhood to adulthood.

## Abstract (ja)

**【背景】:**

出産後も就業を続ける母親が増加していることにより、育児への参加者は多様化してきている。しかしながら、主な養育者による児の生活習慣への影響はあまり明らかでない。そこで本研究では、養育者の違いがその後の児の間食習慣と体格指数（body mass index: BMI）に与える影響を前向きに検討した。

**【方法】:**

IBACHIL研究（The Ibaraki Children’s Cohort Study）は、児が3歳時に保護者が健康アンケートに回答した4,592人を対象としている。その後、6歳時と12歳時に保護者へ、22歳時に本人へ、追跡健康アンケートを実施し、3歳時点での日中の主な養育者ごとに、6歳、12歳、22歳時点での間食習慣と過体重の割合、追跡期間中のBMIの平均値を比較した。

**【結果】:**

母親に育てられた児に比べ、祖父母に育てられた児では、6歳時、12歳時点の夕食前に間食をする割合が男女とも高かった。22歳時の過体重の割合は、母親に育てられた男児の11.2%に比べ、祖父母に育てられた児で18.5%と有意に高かった（*P* = 0.037）が、女児では差はなかった。追跡期間中のBMIの経年平均値は、母親に育てられた児に比べ、祖父母に育てられた児で男女とも高かった（男児：0.47 kg/m^2^、女児：0.35 kg/m^2^）。

**【結論】:**

3歳時に祖父母に養育されることが、幼児期から成人期における間食習慣や過体重、BMIの高値と関連した。

## INTRODUCTION

The participation of women in society as workers has markedly increased throughout the world.^1^ In Japan, the employment rate for women between 25 and 44 years of age has gradually increased since 1991.^2^ The number of women who continue to work after the termination of their childcare leave has also increased from 9.3% in 1985 to 24.2% in 2009. Among women with children over the age of 1 year, 96.6% of mothers who continue to work use parental support and childcare facilities, such as a nursery school.^3^

Consequently, types of participants in childcare, including grandparents and nursery schools, have become diversified. Subsequent dietary habits may be influenced by the feeding practices used by such diverse caregivers during children’s upbringing.^4^^,^^5^ In particular, the grandparent-child relationship is important, because parenting methods are transmitted across generations,^6^ and grandparents are one of the most stable relationships in children’s lives as primary caregivers.^7^ For example, 52.3% of grandmothers in Japan provided care for their grandchildren until their first grandchild became 3 years old.^3^ The proportion of mothers who used nursery school until their children became 3 years old increased from 18.9% to 34.9% between 1990 and 1994.^3^ There is some evidence that parenting method affects children’s eating habits.^8^^–^^10^ A cross-sectional study reported that children aged 8 to 10 years cared for by grandparents had higher consumption of unhealthy snacks and sugar-added drinks and were twice as likely to be overweight compared with those cared for by their parents.^10^ Similarly, a cohort study in the United Kingdom showed that parenting by grandparents during the first 4 months of life was associated with a 34% higher risk of overweight among their children at age 3 years than children who were not parented by grandparents.^11^

However, the impact of the main daytime caregiver on children’s habits afterwards has not been elucidated. In this cohort study, we sought to examine the effect of caregiver differences on subsequent childhood habituation, focusing on between-meal eating habits, being overweight, and body mass index (BMI). Our a priori hypothesis was that children cared for by grandparents at 3 years of age would have more between-meal eating habits, would be more likely to be overweight, and would have higher mean BMI later in life compared to those cared for by their mothers during the same period.

## METHODS

### Study subjects

The Ibaraki Children’s Cohort (IBACHIL) Study is a long-term prospective cohort study involving children born in 1989 in 87 communities of Ibaraki Prefecture, Japan. In 1992, we distributed a health questionnaire to parents at the site of community-based health checkup for 3-year-old children. A total of 4592 out of 10 526 children who had the check-up returned the questionnaires by mail after completing them at home. We excluded 311 children for the following reasons: (1) incomplete caregiver information at the baseline questionnaire (*n* = 18), (2) undetermined gender (*n* = 1), (3) not living with a mother (*n* = 234), and (4) being cared for by their fathers and others (*n* = 71). Ultimately, 4281 children (2239 boys and 2042 girls) were enrolled in this study (response rate, 40.7%). We subsequently carried out follow-up surveys at age 6 (2141 subjects; follow-up rate, 50.0%), 12 (2375 subjects; follow-up rate, 55.5%), and 22 years (1559 subjects; follow-up rate, 36.4%) based on mail-in surveys. At ages 6 and 12 years, the respondents were the parents. At age 22 years, the respondents were the study subjects themselves.

This study protocol was approved by the Epidemiology Combination Ethics Review Committee of Ibaraki Prefecture.

### Baseline measurements

A questionnaire administered to parents regarding their 3-year-old children included questions regarding several lifestyle and physical factors, such as height, weight, eating habits, and living arrangements. The “main daytime caregivers” were discerned from the original questionnaire at 3 years of age, which allowed for classification into (1) mothers, (2) grandparents, and (3) nursery school or kindergarten staff.

The questionnaire asked about between-meal eating (three or more times a day, twice a day, once a day, or no snacking), between-meal eating before bedtime (everyday, three-five times a week, one-two times a week, two-to-three times a month, or once a month), birth weight, types of feeding (maternal breast-feeding, artificial feeding, or mixed feeding), usual wake-up time (before 6 am, between 6 and 7 am, between 7 and 8 am, between 8 and 9 am, or after 9 am), usual bed time (before 8 pm, between 8 pm and 9 pm, between 9 and 10 pm, between 10 and 11 pm, or after 11 pm), physical activity (which parents estimated qualitatively as very active, active, not too active, or not active), major type of playing (outdoor play or indoor play), living with brothers or sisters (yes or no), picky eating (yes or no), father’s type of work (full-time service, part-time job, independent business, agriculture, forestry and fisheries, unemployed, or other), and mother’s type of work (full-time service, part-time job, independent business, agriculture, forestry and fisheries, full-time housewife, unemployed, or other). We defined “between-meal eating three times or more per day” as between-meal eating (yes), “between-meal eating before bedtime every day” as between-meal eating before bedtime (yes), “maternal breast-feeding or mixed feeding” as maternal breast-feeding (yes), “waking up after 9 am” as waking up late (yes), “going to bed after 11 pm (yes)” as going to bed late (yes), “very active or active” as physically active, “outdoor play” as playing outside, “father’s full-time or part-time job” as father’s employment (yes), and “mother’s full-time or part-time job” as mother’s employment (yes). BMI was calculated as weight (kg) divided by the square of the height in meters (m^2^). Overweight was defined as a BMI ≥17.9 kg/m^2^ for boys and ≥17.6 kg/m^2^ for girls.^12^

### Follow-up measurements

Similar questionnaires were distributed when children were aged 6, 12, and 22 years. Between-meal eating habit information was collected with the 6-year-old and 12-year-old follow-up surveys based on the questionnaire administered to parents and with 22-year-old follow-up survey based on the questionnaire administered to the subjects.

From the questionnaire at ages 6 and 12 years, we defined “between-meal eating 30 minutes before dinner” as between-meal eating before dinner (yes), “between-meal eating three times or more per day” as between-meal eating (yes), and “between-meal eating before bedtime three times or more per week” as between-meal eating before bedtime (yes). At age 22 years, the same definition was applied except for between-meal eating, which was defined as “between-meal eating five times or more per week”.

Overweight was defined as a BMI ≥17.6 kg/m^2^ for boys and ≥17.3 kg/m^2^ for girls at the age of 6 years, ≥21.2 kg/m^2^ for boys and ≥21.7 kg/m^2^ for girls at the age of 12 years, and ≥25.0 kg/m^2^ for both boys and girls at the age of 22 years.^12^

For the analyses, we focused on the prevalence of between-meal eating before dinner, being overweight, and BMI level as the primary outcomes. We also used frequent between-meal eating and between-meal eating before bedtime as secondary outcomes.

### Statistical analyses

Differences in mean values or frequencies of the baseline characteristics according to daytime caregivers (grandparents and nursery school or kindergarten staff versus mothers) were tested by the analysis of variance (ANOVA) or chi-square test. Sex-specific odds ratios (ORs) and 95% confidence intervals (95% CIs) for each outcome according to the daytime caregiver were examined using logistic regression models, adjusted for baseline types of feeding, wake-up time, time of sleep, physical activity, playing outside, living with brothers or sisters, picky eating, and father’s employment. To examine whether the caregiver and overweight association was mediated by between-meal eating, we adjusted further for between-meal eating parameters at each age: ‘between-meal eating three times or more per day’ at age 3 years and ‘between-meal eating before dinner’ at ages 6, 12, and 22 years.

To examine the impact of the caregiver on BMI over time, the analysis of repeated measures of BMI was performed using mixed-effects models, which accounted for the correlation existing between repeated measurements taken from the same individual.^13^ In the present study, we used the following model:

$$\begin{array}{l} y_{ij}=(\beta_{0}+\beta_{0i})+\beta_{P}\cdot {\text{Caregiver}}_{ij}+\beta_{T}\cdot {\text{Time}}_{ij}\\ \;\;+\beta_{PT}\cdot {\text{(Caregiver×Time)}}_{ij}+\varepsilon_{ij} \end{array}$$

where *y_ij_* represents the BMI for individual *i* taken at time *j*; *β*_0_ represents the intercept and *β_T_* the slope of the linear relationship between the BMI and time; *β_P_* represents the effect of caregiver type on BMI, considered as constant across time; and *β_PT_* represents the effect of caregiver type on the slope describing the linear relationship between BMI and time. Coefficients for this model were estimated by maximization of the likelihood using the SAS procedure MIXED and specifying a compound symmetry structure for the covariance matrix. For interpretability, exponentiated regression coefficients from regression models with continuous outcomes were reported as differences in BMI associated with caregiver type after adjustment for potential confounding factors. In multivariate analysis, we adjusted for types of feeding, wake-up time, time of going to bed, physical activity, playing outside, living with brothers or sisters, picky eating, and father’s employment, which were significantly associated with caregiver type at baseline. Further, all between-meal eating was adjusted in the way mentioned above.

All statistical analyses were performed with SAS version 9.3 software (SAS Institute, Inc., Cary, NC, USA).^14^ All probability values for statistical tests were two-tailed, and values of *P* < 0.05 was regarded as statistically significant.

## RESULTS

At the age of 3, 75.5% (75.4% in boys and 75.6% in girls) of subjects were cared for by mothers, 10.6% (10.5% in boys and 10.6% in girls) by grandparents, and 13.9% (14.1% in boys and 13.7% in girls) by nursery school or kindergarten staff. As shown in Table 1, the mean baseline BMI, the prevalence of overweight, and the prevalence of between-meal eating were higher among children cared for by grandparents and by nursery school or kindergarten staff for both boys and girls compared to those cared for by mothers. Overall, children cared for by grandparents were more likely to be physically active and to have employed mothers, and they were less likely to be fed by maternal breast milk, wake up late (boys only), play outside, be picky eaters (boys only), and have employed fathers (boys only) compared with those cared for by mothers. Children cared for by nursery school or kindergarten staff were more likely to have employed mothers and less likely to go to bed late (girls only), live with brothers or sisters (girls only), be picky eaters, and have employed fathers compared with those cared for by mothers.

**Table 1.** Sex-specific mean values and proportion of baseline characteristics at age 3 among 2239 boys and 2042 girls, IBACHIL, 1989

Caregivers for 3-year-old children	Boys			Girls		
	
	Mothers	Grandparents	Nursery school or kindergarten staff	Mothers	Grandparents	Nursery school or kindergarten staff
	(*n* = 1693)	(*n* = 233)	(*n* = 313)	(*n* = 1547)	(*n* = 217)	(*n* = 278)
Between-meal eating ≥3 times or more per day, %	13.1	31.4***	17.7*	13.3	28.7***	21.0**
Between-meal eating before bedtime every day, %	11.2	15.0	14.3	13.0	12.3	18.2*
BMI, kg/m^2^	16.2	16.5***	16.4**	16.0	16.4***	16.2**
Overweight, %	6.4	13.8***	11.0**	9.8	15.0*	11.0
Birth weight, g	3230	3174	3245	3123	3179	3116
Maternal breast feeding, %	60.0	47.6***	57.5	61.5	50.0**	58.3
Waking-up after 9 am, %	5.4	1.7**	0	6.6	7.9	0
Going to bed after 11 pm, %	6.0	3.9	3.5	5.9	7.8	2.5*
Physically active, %	91.7	96.1*	94.2	88.7	93.1*	91.0
Playing outside, %	95.5	91.4**	94.9	95.0	91.2*	96.0
Living with brothers or sisters, %	79.9	78.5	75.7	79.8	76.5	70.5***
Picky eating, %	55.5	44.6**	47.9*	50.6	46.0	41.5**
Father’s employment, %	86.4	79.4**	73.8***	85.5	84.1	68.7***
Mother’s employment, %	3.3	87.8***	71.3***	3.3	90.7***	72.7***
Paternal BMI, kg/m^2^	23.1	23.2	23.1	23.1	23.0	23.2
Maternal BMI, kg/m^2^	21.2	21.4	21.2	21.2	21.3	21.0

BMI, body mass index.

*P < 0.05, **P < 0.01, ***P < 0.001, compared with mothers as caregivers.

As shown in Table 2, the multivariable-adjusted ORs of between-meal eating at age 3 years among children cared for by grandparents compared to those cared for by mothers were 2.9 (95% CI, 2.1–4.1) for boys and 2.5 (95% CI, 1.8–3.5) for girls. The multivariable-adjusted ORs of between-meal eating before dinner among children cared for by grandparents compared to those cared for by mothers were 2.1 (95% CI, 1.4–3.1) for boys and 2.5 (95% CI, 1.7–3.8) for girls at age 6 years, 1.3 (95% CI, 0.9–1.8) for boys and 1.9 (95% CI, 1.3–2.8) for girls at age 12 years, and 0.9 (95% CI, 0.6–1.5) for boys and 1.2 (95% CI, 0.7–2.0) for girls at age 22 years. The respective ORs among children cared for by nursery school or kindergarten staff compared to those cared for by mothers were 1.4 (95% CI, 1.0–1.9) for boys and 1.8 (95% CI, 1.3–2.5) for girls at age 3 years, 1.6 (95% CI, 1.1–2.4) for boys and 1.6 (95% CI, 1.0–2.4) for girls at age 6 years, 1.0 (95% CI, 0.7–1.5) for boys and 1.7 (95% CI, 1.1–2.5) for girls at age 12 years, and 1.2 (95% CI, 0.8–1.9) for boys and 0.9 (95% CI, 0.5–1.5) for girls at age 22 years.

**Table 2.** Sex-specific and multivariable-adjusted odds ratios of eating habits according to caregivers

Caregiver for 3-year-old children	Boys				Girls			
	
	Number of subjects	Number of cases	Crude OR	Multivariable- adjusted OR^a^	Number of subjects	Number of cases	Crude OR	Multivariable- adjusted OR^a^
At age 3								
Between-meal eating ≥3 times or more per day								
*Mothers*	1666	218	1.0	1.0	1535	204	1.0	1.0
*Grandparents*	229	72	3.0 (2.2–4.2)***	2.9 (2.1–4.1)***	216	62	2.6 (1.9–3.7)***	2.5 (1.8–3.5)***
*Nursery school or kindergarten staff*	310	55	1.4 (1.0–2.0)*	1.4 (1.0–1.9)	276	58	1.7 (1.3–2.4)***	1.8 (1.3–2.5)***

Between-meal eating before bedtime every day								
*Mothers*	1660	186	1.0	1.0	1512	197	1.0	1.0
*Grandparents*	226	34	1.4 (0.9–2.1)	1.4 (0.9–2.1)	212	26	0.9 (0.6–1.4)	0.9 (0.6–1.4)
*Nursery school or kindergarten staff*	300	43	1.3 (0.9–1.9)	1.3 (0.9–1.9)	274	50	1.5 (1.1–2.1)*	1.6 (1.1–2.3)**

At age 6								
Between-meal eating before dinner								
*Mothers*	792	359	1.0	1.0	712	302	1.0	1.0
*Grandparents*	123	79	2.2 (1.5–3.2)***	2.1 (1.4–3.1)***	115	74	2.5 (1.6–3.7)***	2.5 (1.7–3.8)***
*Nursery school or kindergarten staff*	121	69	1.6 (1.1–2.4)*	1.6 (1.1–2.4)*	109	59	1.6 (1.1–2.4)*	1.6 (1.0–2.4)*

Between-meal eating ≥3 times or more per day								
*Mothers*	803	17	1.0	1.0	715	20	1.0	1.0
*Grandparents*	125	8	3.2 (1.3–7.5)**	3.1 (1.3–7.7)*	116	7	2.2 (0.9–5.4)	2.7 (1.1–6.7)*
*Nursery school or kindergarten staff*	123	5	2.0 (0.7–5.4)	1.9 (0.7–5.4)	111	6	2.0 (0.8–5.1)	2.3 (0.9–6.3)

Between-meal eating before bedtime ≥3 times or more per week								
*Mothers*	802	76	1.0	1.0	714	69	1.0	1.0
*Grandparents*	125	17	1.5 (0.9–2.6)	1.5 (0.8–2.7)	115	14	1.3 (0.7–2.4)	1.4 (0.7–2.5)
*Nursery school or kindergarten staff*	123	12	1.0 (0.5–2.0)	1.1 (0.6–2.0)	110	14	1.4 (0.7–2.5)	1.6 (0.8–3.0)

At age 12								
Between-meal eating before dinner								
*Mothers*	880	483	1.0	1.0	732	376	1.0	1.0
*Grandparents*	165	103	1.4 (1.0–1.9)	1.3 (0.9–1.8)	136	92	2.0 (1.3–2.9)***	1.9 (1.3–2.8)**
*Nursery school or kindergarten staff*	154	88	1.1 (0.8–1.5)	1.0 (0.7–1.5)	128	81	1.6 (1.1–2.4)*	1.7 (1.1–2.5)*

Between meal eating ≥5 times or more per week								
*Mothers*	886	367	1.0	1.0	735	348	1.0	1.0
*Grandparents*	166	68	1.0 (0.7–1.4)	1.0 (0.7–1.4)	138	61	0.9 (0.6–1.3)	0.9 (0.6–1.3)
*Nursery school or kindergarten staff*	159	71	1.1 (0.8–1.6)	1.2 (0.8–1.7)	130	54	0.8 (0.5–1.2)	0.9 (0.6–1.3)

Between meal eating before bedtime ≥3 times or more per week								
*Mothers*	884	83	1.0	1.0	736	54	1.0	1.0
*Grandparents*	163	24	1.7 (1.0–2.7)*	1.5 (0.9–2.5)	136	11	1.1 (0.6–2.2)	1.1 (0.6–2.2)
*Nursery school or kindergarten staff*	159	11	0.7 (0.4–1.4)	0.7 (0.4–1.3)	129	11	1.2 (0.6–2.3)	1.2 (0.6–2.5)

At age 22								
Between-meal eating before dinner								
*Mothers*	529	315	1.0	1.0	503	364	1.0	1.0
*Grandparents*	104	61	1.0 (0.6–1.5)	0.9 (0.6–1.5)	93	70	1.2 (0.7–1.9)	1.2 (0.7–2.0)
*Nursery school or kindergarten staff*	100	64	1.2 (0.8–1.9)	1.2 (0.8–1.9)	84	58	0.9 (0.5–1.4)	0.9 (0.5–1.5)

Between-meal eating ≥5 times or more per week								
*Mothers*	542	141	1.0	1.0	516	203	1.0	1.0
*Grandparents*	107	23	0.8 (0.5–1.3)	0.9 (0.5–1.5)	96	32	0.8 (0.5–1.2)	0.8 (0.5–1.3)
*Nursery school or kindergarten staff*	102	25	0.9 (0.6–1.5)	1.0 (0.6–1.6)	87	35	1.0 (0.7–1.7)	1.1 (0.7–1.8)

Between-meal eating before bedtime ≥3 times or more per week								
*Mothers*	542	109	1.0	1.0	516	50	1.0	1.0
*Grandparents*	108	21	1.0 (0.6–1.6)	1.0 (0.6–1.7)	96	11	1.2 (0.6–2.4)	1.3 (0.6–2.6)
*Nursery school or kindergarten staff*	102	13	0.6 (0.3–1.1)	0.6 (0.3–1.1)	87	10	1.2 (0.6–2.5)	1.3 (0.6–2.8)

OR, odds ratio.

*P < 0.05, **P < 0.01, ***P < 0.001, compared with mothers as caregivers.

^a^Multivariable models adjusted for types of feeding, wake-up time, time to bed, physically active, playing outside, living with brothers or sisters, picky eating, and father’s employment at age 3.

The multivariable-adjusted ORs of those overweight at age 3 years (Table 3) among children cared for by grandparents compared to those cared for by mothers were 2.2 (95% CI, 1.4–3.4) for boys and 1.6 (95% CI, 1.1–2.5) for girls. At age 6 years, the respective ORs among children cared for by grandparents compared to those cared for by mothers were 2.4 (95% CI, 1.4–4.3) for boys and 1.5 (95% CI, 0.8–2.7) for girls. At age 12 years, the respective ORs among children cared for by grandparents compared to those cared for by mothers were 1.9 (95% CI, 1.3–2.8) for boys and 0.9 (95% CI, 0.5–1.6) for girls. The respective ORs at age 22 years among children cared for by grandparents compared to those cared for by mothers were 2.0 (95% CI, 1.1–3.6) for boys and 0.4 (95% CI, 0.1–1.3) for girls. The respective ORs among children cared for by nursery school or kindergarten staff compared to those cared for by mothers were 1.8 (95% CI, 1.2–2.7) for boys and 1.2 (95% CI, 0.8–1.8) for girls at age 3 years, 1.7 (95% CI, 0.9–3.0) for boys and 1.6 (95% CI, 0.9–3.0) for girls at age 6 years, 1.2 (95% CI, 0.8–1.9) for boys and 1.3 (95% CI, 0.8–2.2) for girls at age 12 years, and 1.3 (95% CI, 0.7–2.5) for boys and 1.1 (95% CI, 0.4–2.5) for girls at age 22 years. After further adjustment for the frequency of between-meal eating at each age, the ORs among children cared for by grandparents and by nursery school or kindergarten staff compared to those cared for by mothers did not change.

**Table 3.** Sex-specific and multivariable-adjusted odds ratios of overweight subjects according to caregivers

Caregiver for 3-year-old children	Boys					Girls				
	
	Number of subjects	Number of cases	Crude OR	Multivariable- adjusted OR^a^	Multivariable- adjusted OR^b^	Number of subjects	Number of cases	Crude OR	Multivariable- adjusted OR^a^	Multivariable- adjusted OR^b^
At age 3										
Overweight										
*Mothers*	1647	105	1.0	1.0	1.0	1494	146	1.0	1.0	1.0
*Grandparents*	225	31	2.3 (1.5–3.6)***	2.2 (1.4–3.4)***	2.1 (1.4–3.3)***	207	31	1.6 (1.1–2.5)*	1.6 (1.1–2.5)*	1.6 (1.0–2.5)*
*Nursery school or* *kindergarten staff*	301	33	1.8 (1.2–2.7)**	1.8 (1.2–2.7)**	1.8 (1.2–2.7)**	264	29	1.1 (0.7–1.7)	1.2 (0.8–1.8)	1.2 (0.7–1.8)

At age 6										
Overweight										
*Mothers*	588	68	1.0	1.0	1.0	507	75	1.0	1.0	1.0
*Grandparents*	90	20	2.2 (1.3–3.8)**	2.4 (1.4–4.3)**	2.4 (1.3–4.3)**	86	18	1.5 (0.9–2.7)	1.5 (0.8–2.7)	1.4 (0.8–2.6)
*Nursery school or* *kindergarten staff*	97	16	1.5 (0.8–2.7)	1.7 (0.9–3.0)	1.6 (0.9–3.0)	81	17	1.5 (0.9–2.8)	1.6 (0.9–3.0)	1.6 (0.9–3.0)

At age 12										
Overweight										
*Mothers*	856	140	1.0	1.0	1.0	712	100	1.0	1.0	1.0
*Grandparents*	162	45	2.0 (1.3–2.9)***	1.9 (1.3–2.8)**	1.9 (1.3–2.8)**	129	17	0.9 (0.5–1.6)	0.9 (0.5–1.6)	1.0 (0.6–1.7)
*Nursery school or* *kindergarten staff*	149	29	1.2 (0.8–1.9)	1.2 (0.8–1.9)	1.1 (0.7–1.8)	127	22	1.3 (0.8–2.1)	1.3 (0.8–2.2)	1.3 (0.8–2.3)

At age 22										
Overweight										
*Mothers*	544	61	1.0	1.0	1.0	511	36	1.0	1.0	1.0
*Grandparents*	108	20	1.8 (1.0–3.1)*	2.0 (1.1–3.6)*	2.0 (1.1–3.6)*	95	3	0.4 (0.1–1.4)	0.4 (0.1–1.3)	0.4 (0.1–1.4)
*Nursery school or* *kindergarten staff*	102	14	1.3 (0.7–2.4)	1.3 (0.7–2.5)	1.3 (0.7–2.5)	85	7	1.2 (0.5–2.8)	1.1 (0.4–2.5)	1.1 (0.5–2.5)

OR, odds ratio.

*P < 0.05, **P < 0.01, ***P < 0.001, compared with mothers as caregivers.

^a^Multivariable models adjusted for types of feeding, wake-up time, time to bed, physically active, playing outside, living with brothers or sisters, picky eating, and father’s employment at age 3.

^b^Multivariate models adjusted further for between-meal eating; ‘between-meal eating ≥3 times per day’ for the analysis at age 3, and ‘between-meal eating before dinner’ for the analyses at ages 6, 12, and 22.

Crude and multivariable-adjusted differences in BMI (repeated across all time points) associated with caregiver type are presented in Table 4. Children cared for by grandparents had higher BMI over time than those cared for by mothers (coefficient = 0.47 [95% CI, 0.24–0.70] for boys and 0.35 [95% CI, 0.13–0.57] for girls), and these differences did not change after adjusting for confounding variables (0.42 [95% CI, 0.19–0.66] for boys and 0.36 [95% CI, 0.14–0.58] for girls). Children cared for by nursery school or kindergarten staff had higher BMI than those cared for by mothers (0.32 [95% CI, 0.11–0.53] for boys and 0.26 [95% CI, 0.06–0.47] for girls), and these differences did not change after adjusting for confounding variables (0.27 [95% CI, 0.06–0.48] for boys and 0.27 [95% CI, 0.06–0.48] for girls). After further adjustment for the frequency of between-meal eating at each age, the results still did not change.

**Table 4.** Sex-specific and multivariable-adjusted difference for BMI changes overtime according to caregivers by mixed effect model

Caregivers for 3-year-old children	Boys			Girls		
	
	Mothers	Grandparents	Nursery school or kindergarten staff	Mothers	Grandparents	Nursery school or kindergarten staff
	(*n* = 1693)	(*n* = 233)	(*n* = 313)	(*n* = 1547)	(*n* = 217)	(*n* = 278)
Crude	Reference	0.47 (0.24–0.70)	0.32 (0.11–0.53)	Reference	0.35 (0.13–0.57)	0.26 (0.06–0.47)
Multivariable-adjusted^a^	Reference	0.42 (0.19–0.66)	0.27 (0.06–0.48)	Reference	0.36 (0.14–0.58)	0.27 (0.06–0.48)
Multivariable-adjusted^b^	Reference	0.42 (0.19–0.66)	0.27 (0.06–0.48)	Reference	0.33 (0.10–0.55)	0.25 (0.04–0.46)
Multivariable-adjusted^c^	Reference	0.43 (0.20–0.67)	0.24 (0.03–0.46)	Reference	0.36 (0.13–0.58)	0.25 (0.04–0.46)
Multivariable-adjusted^d^	Reference	0.43 (0.19–0.67)	0.27 (0.05–0.48)	Reference	0.36 (0.14–0.59)	0.27 (0.06–0.48)
Multivariable-adjusted^e^	Reference	0.45 (0.21–0.68)	0.27 (0.06–0.49)	Reference	0.37 (0.14–0.59)	0.27 (0.06–0.48)

BMI, body mass index.

^a^Multivariable models adjusted for types of feeding, wake-up time, time to bed, physically active, playing outside, living with brothers or sisters, picky eating, and father’s employment at age 3.

^b^Adjusted further for between-meal eating ≥3 times or more per day at age 3.

^c^Adjusted further for between-meal eating before dinner at age 6.

^d^Adjusted further for between-meal eating before dinner at age 12.

^e^Adjusted further for between-meal eating before dinner at age 22.

## DISCUSSION

In this prospective study, we found that children cared for by grandparents in early childhood had a higher proportion of between-meal eating habits for both genders in childhood and adolescence, a higher proportion of overweight for boys, and a higher mean BMI for both genders from childhood to adulthood. Similar but weaker trends were observed for children cared for by nursery school or kindergarten staff. To our knowledge, this is the first prospective study to track such an association from childhood to adulthood.

Parenting style has been reported to affect children’s eating habits.^4^^,^^5^ Family food environments have been shown to be associated with children’s dietary habits of consuming sweet snacks and high-energy drinks, which are likely to promote fatness.^15^ Parenting attitudes, such as parental forcefulness or encouragement to eat, and food offers have also been associated with children’s relative weight.^16^ A cross-sectional study of elementary and junior high school children in the Tokushima Prefecture Study reported that eating meals with family every day was associated with a lower prevalence of obesity, defined as more than 120% of the gender- and height-specific standard weight, as well as with desirable habits, such as eating balanced meals.^17^ The Toyama Birth Cohort Study showed that the OR of irregular snack intake was 0.63 (95% CI, 0.55–0.73) among children cared for by mothers compared to those cared for by others, but there was no association between caregiver type and obesity (Kaup Index; BMI >18.0) at age 3 years.^18^

The prevalence of overweight and mean BMI pooled over multiple measures across time were higher among boys cared for by grandparents than those cared for by mothers. For girls, we found a similar association between caregiver type and mean BMI, but not between caregiver type and prevalence of overweight, probably because the prevalence of overweight was lower in girls (6.7%) compared with that in boys (12.6%). Further, overweight girls cared for by grandparents were less likely to participate in the follow-up survey at age 22 years because the follow-up rate at age 22 years was the lowest among girls whose BMI was in the highest quartile at age 12 years (49.5% for the highest quartile, 63.3% for the third quartile, 62.9% for the second quartile, and 70.8% for the lowest quartile of BMI). No such difference was observed for boys (respective follow-up rates of 55.0%, 46.9%, 58.8%, and 51.6% for highest to lowest quartiles).

The advantage of this study is that the long-term prospective design made it possible to observe prolonged lifestyle habits from ages 3 to 22 years. To our knowledge, this is the first longitudinal study to examine the association between grandparent-grandchildren relationships and outcomes of eating habits and being overweight.

On the other hand, the present study had several limitations. First, information such as height, weight, and caregiver type were derived from self-administered questionnaires. According to the Japan Public Health Center-based Prospective Study, BMIs, as determined by self-administered questionnaires, are slightly lower than the actual measured values; however, the Spearman rank-correlation coefficient was approximately 0.9 in both men and women.^19^ Therefore, assuming that self-reported BMIs in this study were accurate, the misclassification of those in the overweight category may be small. Second, the follow-up rate was not high (36.4% at age 22 years), which may be due to participants moving to other prefectures to seek higher education or occupation. Therefore, the impact of follow-up bias should be taken into consideration. However, among children cared for by mothers, the proportion of between-meal eating at baseline was higher in responders than in non-responders at age 6 years (14.8% versus 11.8%, *P* = 0.01), at age 12 years (15.8% versus 10.6%, *P* < 0.001), and at age 22 years (14.8% versus 12.4%, *P* = 0.06). There were no marked differences in the proportions of overweight at age 3 years between responders and non-responders at age 6 years (7.9% versus 7.7%, *P* = 0.91 for boys and 9.9% versus 11.0%, *P* = 0.39 for girls), at age 12 years (7.5% versus 8.1%, *P* = 0.58 for boys and 10.5% versus 10.4%, *P* = 0.95 for girls), and at age 22 years (7.3% versus 8.0%, *P* = 0.52 for boys and 10.4% versus 10.5%, *P* = 0.91 for girls). Among children cared for by mothers, the differences in the prevalence of overweight were less prominent in responders than in non-responders at age 6 years (10.0% versus 9.5%, *P* = 0.73 for boys and 8.7% versus 10.8%, *P* = 0.17 for girls), at age 12 years (9.3% versus 10.3%, *P* = 0.47 for boys and 10.1% versus 9.5%, *P* = 0.69 for girls), and at age 22 years (10.8% versus 9.3%, *P* = 0.33 for boys and 9.7% versus 9.8%, *P* = 0.91 for girls). With respect to children cared for by grandparents and by nursery school or kindergarten staff, these proportions were similar between respondents and non-respondents. Therefore, it is unlikely that the higher proportions of between-meal eating and overweight in children cared for by grandparents and by nursery school or kindergarten staff compared to those cared for by mothers were due to follow-up bias. Third, detailed information on diets was not collected, so total energy from the diet and energy from between-meal eating were unknown. Fourth, we did not collect information on parental smoking status or parental education. However, those factors are unlikely to be confounders. Parental smoking may lead to children’s increased BMI and overweight,^20^ but little evidence has supported the association between parental smoking and having grandparents as caregivers. Low parental education also may lead to children’s increased BMI and overweight,^21^ but little evidence supports the association between low mother’s education and having grandparents as caregivers. Finally, it is uncertain whether our results were generalizable to other countries because the relationship between grandparents and children may vary among countries. However, the present findings may be applicable to some other countries, such as China,^8^^,^^10^ where grandparents tend to spoil their grandchildren by allowing between-meal eating.

The public health perspectives of this study warrant discussion. Our findings suggest that grandparents’ method of caring for their grandchildren contributes to the establishment of grandchildren’s habits of between-meal eating and becoming overweight. However, in the present study, we found no robust evidence that the caregiver-overweight association was mediated by between-meal eating habits. Further, while a cross-sectional study indicated that snacking habit was associated with higher prevalence of overweight in children,^22^ several other prospective studies showed that snacking habits were not associated with risk of weight gain in children and adolescents.^23^^,^^24^ Physical inactivity and picky eating may be potential mediators; however, our results did not support this inference, because children cared for by grandparents were more physically active, despite playing outside less than those cared for by mothers, and were less likely to be picky eaters compared with those cared for by mothers. Although we had no relevant data in the present study, high total energy intake may be a potential mediator. According to a descriptive study conducted in China,^8^ grandparents were more likely to urge their grandchildren to eat more at meals and serve larger portions, which may lead to a high energy intake and children’s overweight.

In conclusion, being cared for by grandparents and by nursery school or kindergarten staff was associated with between-meal eating habits in childhood and adolescence, as well as with being overweight and having increased BMI from childhood to adulthood compared to being cared for by mothers.

## ONLINE ONLY MATERIAL

Abstract in Japanese.
