# Supplementary material for: Impact of Caregiver Type for 3-Year-Old Children on Subsequent Between-Meal Eating Habits and Being Overweight From Childhood to Adulthood: A 20-Year Follow-up of the Ibaraki Children’s Cohort (IBACHIL) Study
Source: J Epidemiol. 2015 Sep 5;25(9):600–7. doi: 10.2188/jea.JE20140078 (PMC4549612; doi:10.2188/jea.JE20140078)
Supplement: Abstract in Japanese. [file je-25-600-s001.pdf]

3 歳時における養育者の違いが，幼児期から成人期までのその後の児の間食習慣と過体重に与える影響：Ibaraki Children's Cohort (IBACHIL) Study による 20 年間の追跡研究

佐田みずき[1,2]、山岸良匡[2,3]、西連地利己[2,4]、池田愛[5]、入江ふじこ[6,*]、渡辺宏[7]、磯博康[1]、大田仁史[2]

1　大阪大学大学院医学系研究科社会医学講座公衆衛生学
2　公益財団法人　茨城県立総合健診協会　茨城県立健康プラザ
3　筑波大学医学医療系社会健康医学
4　獨協医科大学医学部公衆衛生学講座
5　順天堂大学大学院医学研究科公衆衛生学講座
6　茨城県保健福祉部保健予防課
7　公益財団法人　茨城県総合健診協会

【背景】

出産後も就業を続ける母親が増加していることにより、育児への参加者は多様化してきている。しかしながら、主な養育者による児の生活習慣への影響はあまり明らかでない。そこで本研究では、養育者の違いがその後の児の間食習慣と体格指数（body mass index: BMI）に与える影響を前向きに検討した。

【方法】

IBACHIL 研究（The Ibaraki Children's Cohort Study）は、児が 3 歳時に保護者が健康アンケートに回答した 4,592 人を対象としている。その後、6 歳時と 12 歳時に保護者へ、22 歳時に本人へ、追跡健康アンケートを実施し、3 歳時点での日中の主な養育者ごとに、6 歳、12 歳、22 歳時点での間食習慣と過体重の割合、追跡期間中の BMI の平均値を比較した。

【結果】

母親に育てられた児に比べ、祖父母に育てられた児では、6 歳時、12 歳時点の夕食前に間食をする割合が男女とも高かった。22 歳時の過体重の割合は、母親に育てられた男児の 11.2%に比べ、祖父母に育てられた児で 18.5%と有意に高かった（$P = 0.037$）が、女児では差はなかった。追跡期間中の BMI の経年平均値は、母親に育てられた児に比べ、祖父母に育てられた児で男女とも高かった（男児：0.47 kg/m^2、女児：0.35 kg/m^2）。

【結論】

3 歳時に祖父母に養育されることが、幼児期から成人期における間食習慣や過体重、BMI の高値と関連した。

キーワード：子ども、食習慣、過体重、コホート研究、疫学
